# Draft Genome Sequence of the Type Strain *Pseudomonas jessenii* DSM 17150

**DOI:** 10.1128/genomeA.01035-17

**Published:** 2017-09-28

**Authors:** Ewa M. Furmanczyk, Michal A. Kaminski, Andrzej Dziembowski, Leszek Lipinski, Adam Sobczak

**Affiliations:** a Institute of Biochemistry and Biophysics, Polish Academy of Sciences, Warsaw, Poland; b Institute of Genetics and Biotechnology, Faculty of Biology, University of Warsaw, Warsaw, Poland

## Abstract

We present the draft genome sequence of *Pseudomonas jessenii* type strain DSM 17150. The assembly consists of 13 contigs, contains 6,537,206 bp, and has a GC content of 59.7%.

## GENOME ANNOUNCEMENT

*Pseudomonas* is the largest genus of Gram-negative bacteria and includes microorganisms that degrade a wide range of substrates, including amino acids, alcohols, and fatty acids and also xenobiotics like polyaromatic hydrocarbons. Members of *Pseudomonas* identified in soil, active sludge, plant surfaces, and freshwater or marine environments play important roles in the global carbon and nitrogen cycle.

Here, we present the draft genome sequence of *Pseudomonas jessenii* type strain DSM 17150 (equivalent to CIP 105274 = CCM 4840 = CFML 95-307), which was isolated from natural mineral water (1) and belongs to the *Pseudomonas jessenii* subgroup inside the *Pseudomonas fluorescens* lineage.

Genomic DNA was isolated using bead beating and a modified protocol from Zhou et al. (2). Briefly, a bacterial pellet from 5 ml of overnight culture was resuspended in 2 ml Zhou buffer; 0.5 g of 0.5-mm zirconia-silica beads was added, and the mixture was lysed by vortexing for 2 × 30 s at 3,200 rpm. The supernatant collected in a fresh tube was treated with 30 μl of lysozyme (100 mg/ml), 10 μl of achromopeptidase (100 kU/ml), and 8 μl RNase A (10 mg/ml), and then incubated for 1 h at 37°C. Next, 8 μl of proteinase K (40 mg/ml) was added to the supernatant and the sample was further incubated for 30 minutes at 37°C. Proteins in the sample were precipitated by the addition of 0.25 ml of 20% SDS to the mixture, incubated 1 hour at 55°C with mixing, chilled on ice for 2 minutes, and centrifuged (20 minutes at 8,000 rpm). The resulting DNA-containing supernatant was transferred to a fresh tube and extracted twice with chloroform, followed by isopropanol DNA precipitation. The DNA was resuspended in 100 µl of water. Illumina paired-end (insert size of 300 bp) and Nextera mate-pair libraries (insert size of 8 kb) were prepared according to the manufacturers’ protocols (a KAPA HTP DNA library preparation kit for Illumina and a Nextera mate-pair sample prep kit, respectively). Sequencing on an Illumina MiSeq platform (2 × 300 bp) resulted in 752,032 paired reads (paired end) and 1,532,388 paired reads (mate pair). Reads from a paired-end library were processed as follows: adapters were removed with cutadapt script (3) and filtered by length (>100) and quality (q30) (4), and only paired reads were used for assembly. The mate-pair reads were processed with NxTrim (5), and only real mate-pair reads were used for the assembly with SPAdes 3.9.0 (6). Contigs longer than 1 kb were deposited in GenBank and annotated using NCBI Prokaryotic Genome Annotation Pipeline (PGAP) (7). No plasmid sequence was detected in the draft genome.

The assembly consists of 13 contigs, containing 6,537,206 bp, with a GC content of 59.7%. The DSM 17150 genome encodes 6,019 predicted genes, of which 5,826 are protein coding, as well as 72 RNA genes, 59 tRNAs, 9 rRNAs, 4 noncoding RNAs (ncRNAs), and 121 pseudogenes.

The genome contains genes involved in decomposition of crude oil components like toluene (*sdhCDAB*) and benzoate (like *pca* or *benABCD* genes) (8), but also a *copABCD* cluster responsible for copper homeostasis that is possibly involved in copper resistance mechanisms in bacteria. Despite the need to confirm the biochemical properties of the *Pseudomonas jessenii* type strain, the genome sequence presented here will be helpful in future research.

### Accession number(s).

This whole-genome shotgun project has been deposited at DDBJ/ENA/GenBank under the accession no. NIWT00000000. The version described in this paper is the first version, NIWT01000000.

## References

[1] Verhille S, Baida N, Dabboussi F, Izard D, Leclerc H 1999 Taxonomic study of bacteria isolated from natural mineral waters: proposal of *Pseudomonas jessenii* sp. nov. and *Pseudomonas mandelii* sp. nov. Syst Appl Microbiol 22:45–58. doi:10.1016/S0723-2020(99)80027-7. 10188278

[2] Zhou J, Bruns MA, Tiedje JM 1996 DNA recovery from soils of diverse composition. Appl Environ Microbiol 62:316–322. 8593035 10.1128/aem.62.2.316-322.1996 PMC167800

[3] Martin M 2011 Cutadapt removes adapter sequences from high-throughput sequencing reads. EMBnet.journal 17:10. doi:10.14806/ej.17.1.200.

[4] Joshi NA, Fass JN 2011 Sickle: a sliding-window, adaptive, quality-based trimming tool for FastQ files (version 1.33) (software). Available at https://github.com/najoshi/sickle.

[5] O’Connell J, Schulz-Trieglaff O, Carlson E, Hims MM, Gormley NA, Cox AJ 2015 NxTrim: optimized trimming of Illumina mate pair reads. Bioinformatics 31:2035–2037. doi:10.1093/bioinformatics/btv057. 25661542

[6] Bankevich A, Nurk S, Antipov D, Gurevich AA, Dvorkin M, Kulikov AS, Lesin VM, Nikolenko SI, Pham S, Prjibelski AD, Pyshkin AV, Sirotkin AV, Vyahhi N, Tesler G, Alekseyev MA, Pevzner PA 2012 SPAdes: a new genome assembly algorithm and its applications to single-cell sequencing. J Comput Biol 19:455–477. doi:10.1089/cmb.2012.0021. 22506599 PMC3342519

[7] Tatusova T, DiCuccio M, Badretdin A, Chetvernin V, Nawrocki EP, Zaslavsky L, Lomsadze A, Pruitt KD, Borodovsky M, Ostell J 2016 NCBI prokaryotic genome annotation pipeline. Nucleic Acids Res 44:6614–6624. doi:10.1093/nar/gkw569. 27342282 PMC5001611

[8] Das D, Baruah R, Sarma Roy A, Singh AK, Deka Boruah HP, Kalita J, Bora TC 2015 Complete genome sequence analysis of *Pseudomonas aeruginosa* N002 reveals its genetic adaptation for crude oil degradation. Genomics 105:182–190. doi:10.1016/j.ygeno.2014.12.006. 25546474

